# Effect of traditional Chinese medicine on postoperative depression of breast cancer: a systematic review and meta-analysis

**DOI:** 10.3389/fphar.2023.1019049

**Published:** 2023-06-23

**Authors:** Yan Wang, Suying Liu, Ying Zhang, Guanghui Zhu, Heping Wang, Bowen Xu, Yi Xie, Shuhan Yang

**Affiliations:** Department of Oncology, Guang’anmen Hospital, China Academy of Chinese Medical Sciences, Beijing, China

**Keywords:** traditional Chinese medicine, postoperative, depression, breast cancer, meta-analysis

## Abstract

**Background:** Depression is one of the common complications in patients with postoperative breast cancer (BC). Conventional therapies for postoperative depression of BC always have modest treatment outcomes and undesirable side effects. Clinical practice and many studies have shown that traditional Chinese medicine (TCM) has a good effect on postoperative depression of BC. This meta-analysis aimed to assess the clinical effect of TCM as an add-on treatment for postoperative depression of BC.

**Methods:** A systematic and thorough search was conducted on eight online electronic databases up to 20 July 2022. The control group received conventional therapies, and intervention groups received what control groups received plus TCM treatment. Review Manager 5.4.1 was used for statistical analysis.

**Results:** Nine RCTs involved 789 participants who met the inclusion standards. The results showed the intervention group was better at decreasing the score of the Hamilton rating scale for depression (HAMD) (mean difference, MD = −4.21, 95% CI −5.54 to −2.88) and the self-rating depression scale (SDS) (MD = −12.03, 95% CI −15.94 to −8.13), improving clinical efficacy (RR = 1.25, 95% CI 1.14–1.37), increasing the levels of 5-hydroxytryptamine (5-HT) (MD = 0.27, 95% CI 0.20–0.34), dopamine (DA) (MD = 26.28, 95% CI 24.18–28.77), and norepinephrine (NE) (MD = 11.05, 95% CI 8.07–14.04), and influencing the immune index, including the levels of CD3^+^ (MD = 15.18, 95% CI 13.61–16.75), CD4^+^ (MD = 8.37, 95% CI 6.00–10.74), and CD4^+^/CD8^+^ (MD = 0.33, 95% CI 0.27–0.39). The level of CD8^+^ (MD = −4.04, 95% CI −11.98 to 3.99) had no obvious difference between the two groups.

**Conclusion:** The meta‐analysis stated that a therapeutic regimen involving TCM could better improve the depression status in postoperative BC.

## Introduction

According to the global cancer statistics data in 2020, breast cancer (BC) has replaced lung cancer as the most common cancer globally, and its incidence and mortality are the first in female individuals (Sung et al., 2021). Currently, the primary treatment of BC is a comprehensive treatment based on surgery (Miller et al., 2009; Gradishar et al., 2022). Due to the absence of breasts, damaged appearance, and weakened femininity, BC patients will have a sense of shame, which increases the psychological burden of patients and is more likely to cause negative emotions of depression and pessimism after surgery (Xie and He, 2017). In addition, sexual dysfunction, side-effects of radiotherapy and chemotherapy, endocrine disorders, economic burden, and the disease itself will affect the mood of patients, leading to postoperative depression and even suicidal tendencies (Abdollahzadeh et al., 2014; Wang et al., 2015; Grusdat et al., 2022). Many studies have shown that patients suffering from BC have a significantly higher incidence of depression than other cancer populations (Shang et al., 2019; Padmalatha et al., 2021). A systematic review showed that the prevalence of long-term symptoms of depression after BC treatment ranged from 9.4% to 66.1%, and the overall percentage was 39.9% (Maass et al., 2015). There was a study report that the prevalence of depression after mastectomy in BC patients is 64% (Kamińska et al., 2015). An estimate of 51.4% of BC patients after mastectomy exhibited symptoms of depression in the first year (de Raaff et al., 2016).

Depression and these accompanying symptoms not only directly reduce the quality of life of patients but also negatively affect the immune function of patients (Abu-Helalah et al., 2014; Kim et al., 2018; Zhu et al., 2022). The immune system may also play a key role in the link between depression and cancer, although this relationship is currently unclear (Fraile-Martinez et al., 2022). In addition, patients with poor psychological status will decline treatment compliance and survival time (Kim et al., 2022). Current research showed that monoamine neurotransmitters, including 5-hydroxytryptamine (5-HT), dopamine (DA), and norepinephrine (NE), are decreased in patients with depression (Yang et al., 2020). Therefore, selective serotonin reuptake inhibitors, selective serotonin–norepinephrine reuptake inhibitors, and selective serotonin–norepinephrine dopamine reuptake inhibitors were commonly used in increasing the levels of 5-HT, DA, and NE to treat depression (Davies et al., 2019). However, conventional therapies for postoperative depression of BC always have modest treatment outcomes and are often accompanied by autonomic nervous system, gastrointestinal, and nervous system side effects (Carvalho et al., 2014; Tian et al., 2020). A growing body of research showed that the use of syndrome differentiation and treatment under the guidance of traditional Chinese medicine (TCM) theory has achieved good clinical efficacy in the postoperative depression of BC and can improve the depression status, increasing the levels of 5-HT, DA, and NE in the serum, and influence the immune index (Liu and Xiao, 2011; Xiong et al., 2021; Chen et al., 2022).

However, there is no systematic review of TCM efficacy in treating postoperative depression of BC. This study conducted a meta-analysis of the RCTs evaluating the clinical efficacy of TCM in the treatment of patients with postoperative depression of BC and synthesized evidence from the perspective of evidence-based medicine.

## Methods

### Search strategy

We searched the following databases comprehensively from the inception of databases to 20 July 2022: PubMed, Cochrane Library, Embase, Web of Science, China National Knowledge Infrastructure, Wanfang database, VIP database, and Chinese BioMedical Literature database. Medical subject headings (MeSH) terms and free text terms were used to obtain more comprehensive studies. The MeSH terms of “medicine, Chinese traditional,” “botanical drugs,” “breast neoplasms,” “postoperative period,” “depression,” and “depressive disorder” were used to construct search strategies. The search strategy of PubMed is shown in Supplementary Material S2.

### Eligibility criteria

#### Inclusion criteria

1. Patients who were diagnosed with BC by histopathology and underwent surgical treatment; whether or not combined with other adjuvant therapy such as chemotherapy.
2. Only RCTs were included.
3. All patients included were diagnosed with depression diagnostic criteria, including Chinese Classification and Diagnostic Criteria for Mental Disorders Version 3 (CCMD-3); and Diagnostic and Statistical Manual of Mental Disorders, United States.
4. The control group received conventional therapies (e.g., chemoradiotherapy, endocrine therapy, antidepressant drugs, and psychological counseling), and intervention groups received what control groups received plus TCM treatment, including Chinese medicine decoction and Chinese patent medicine.
5. The outcomes included Hamilton’s depression scale (HAMD), depression self-rating score values (SDS), clinical efficacy, and levels of laboratory tests: 5-HT, DA, and NE in the serum, and immune function index (CD3^+^, CD4^+^, CD8^+^, and CD4^+^/CD8^+^).

#### Exclusion criteria

1. Repeated publication;
2. Outcome of interest not included;
3. Original data cannot be obtained by contacting the original author;
4. The language of publication was neither English nor Chinese.

### Study selection and data extraction

EndNote 20 was used to manage the literature. Two researchers (YW and SYL) independently retrieved the titles and abstracts of all articles. Any disagreement in the screening process was consulted with another researcher (YZ) to make a decision. The relevant information was independently extracted and cross-checked by two researchers (YW and SYL) independently, which included: 1) basic information on the article: author’s name, year of publication, study type, and sample size; 2) patient characteristics: age, disease course, operation types, pathologic types, and stage; and 3) treatment outcomes: clinical intervention, intervention time, and outcomes. Disagreements were solved by discussion or consulting a third-party opinion (YZ), imputing a change-from-baseline standard deviation (SD) and mean using a correlation coefficient. A SD of the change from baseline for the experimental intervention was input using the following formula:

SD _E, change_ = √[SD^2^_E, baseline_ + SD^2^_E, final_ - (2 × Corr × SD _E, baseline_ × SD _E, final_)]; Corr = 0.75 (Zhang and Han, 2009).

The mean value of the change from baseline for the experimental intervention was input using the following formula:

Mean _E, change_ = Mean _E, final_−Mean _E, baseline_ (Higgins et al., 2022).

All data were rounded to two decimal places.

### Outcomes of interest

The primary outcome of this study was depression status, evaluated with HAMD or SDS. HAMD was developed in 1960 and is the most commonly used scale in the clinical evaluation of depression. The SDS was developed in 1965, and it is one of the scales recommended by the United States Department of Education, Health, and Welfare for psychopharmacological research. The secondary outcomes included clinical efficacy, levels of laboratory tests (5-HT, DA, and NE) in the serum, and the immune function index of CD3^+^, CD4^+^, CD8^+^, and CD4^+^/CD8^+^. Clinical efficacy evaluation criteria: HAMD or SDS reduction rate = [(pre-treatment score−post-treatment score)/pre-treatment score × 100%]. The HAMD or SDS score reduction rate ≥ 75% was considered as recovery, 50% ∼ 74% as a significant improvement, 25% ∼ 49% as improvement, and <25% as no improvement. Clinical efficacy rate = (recovery + significant effect + improvement)/n × 100% (Zhang, 1998).

### Quality assessment

Two researchers (YW and SYL) used the Cochrane Risk of Bias Tool (RoB) (Higgins et al., 2011) to evaluate the methodological quality of all included RCTs independently. The following seven domains were assessed: random sequence generation, allocation concealment, blinding of participants and personnel, blinding of outcome assessment, incomplete outcome data, selective reporting, and other biases. The included RCTs were assessed as low, uncertain, or high risk of bias. The results are shown in the RoB graph.

### Statistical analysis

Review Manager software (version 5.4.1) was used to perform the meta-analysis. The random-effect model was used to synthesize evidence. Sensitivity or subgroup analysis was conducted to determine the cause of heterogeneity if it exists. The method of deleting studies one by one needed to be used to perform a sensitivity analysis of the results to ensure stability. The subgroup analysis of the meta-analysis results for each outcome was required. The subgroup only includes items related to the comparison. Subgroup analysis was performed based on whether to receive chemotherapy. For continuous variables, effect estimates were calculated as mean difference (MD); and for dichotomous variables, the risk ratio (RR) was calculated. The effect estimates with their 95% confidence intervals (CI) were presented in the forest plots. If meta-analysis was not suitable, descriptive analysis was performed. A funnel plot was used to analyze potential publication bias. *p* < 0.05 was considered statistically significant.

## Results

**FIGURE 1:**
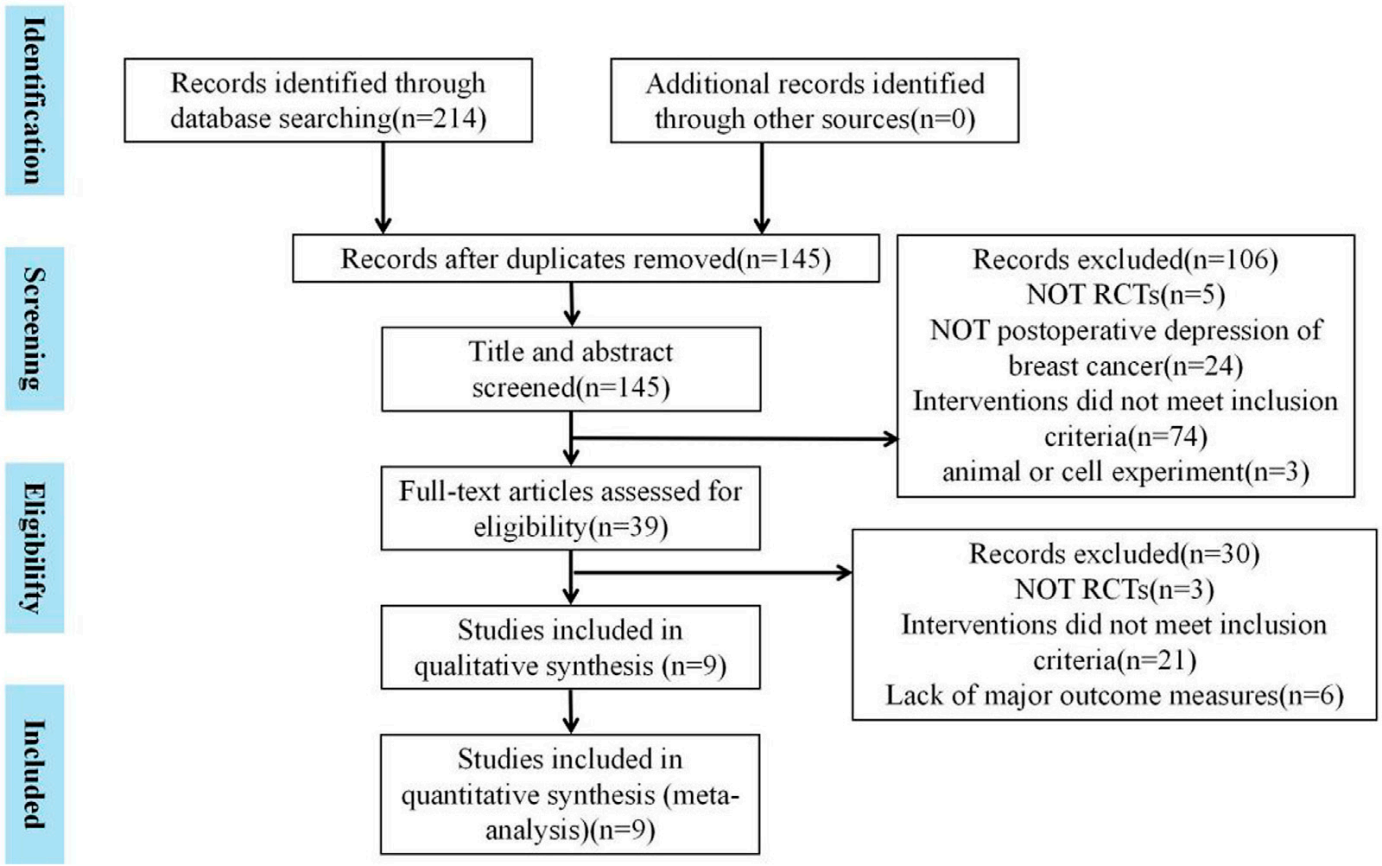
Specific retrieval process is summarized in a flow diagram.

A total of 214 articles were obtained by searching the database. In total, 69 duplicate literature reports were found. After reading the title and abstract, 106 articles were excluded. Then, after strict literature screening and reading the full-text articles according to the inclusion and exclusion criteria, 30 articles were eliminated for the following reasons: 3 did not have RCTs, 21 did not meet the inclusion criteria, and 6 studies lacked outcome measures. Finally, a total of nine eligible trials were included. The specific retrieval process is shown in Figure 1.

### Study characteristics

Nine RCTs were included with 789 participants that were divided into two groups; the control group received conventional therapies (*n* = 389), and intervention groups received what control groups received plus TCM treatment (*n* = 400) (Gong and Jiang, 2015; Zhang et al., 2015; Sun et al., 2016; Chang et al., 2017; Chen, 2017; Wang et al., 2019; Jin et al., 2020; Liu et al., 2020; Xiao, 2021). Study characteristics of included studies are shown in Table 1. Among participants, the average age of patients in the nine studies was between 45 and 54 years old, and the average course of disease ranged from 1 month to more than 5 years. Three studies mentioned the type of surgery (Gong and Jiang, 2015; Chen, 2017; Wang et al., 2019) and the pathological type (Chang et al., 2017; Chen, 2017; Xiao, 2021), but only one study showed that all patients included underwent modified radical operation and had infiltrating ductal carcinoma (Chen, 2017). Five studies involved the stage of disease [(Chang et al., 2017; Chen, 2017), (Liu et al., 2020), (Zhang, 1998; Higgins et al., 2011; Gong and Jiang, 2015; Sun et al., 2016; Chang et al., 2017; Chen, 2017; Wang et al., 2019; Jin et al., 2020; Liu et al., 2020; Xiao, 2021)], in which two RCTs indicated that all patients were in stage II–III (Chang et al., 2017; Chen, 2017). In clinical intervention, four studies mentioned combined chemotherapy (Sun et al., 2016; Chang et al., 2017; Jin et al., 2020; Liu et al., 2020), three studies combined psychological counseling (Chang et al., 2017; Jin et al., 2020; Liu et al., 2020), three studies combined fluoxetine hydrochloride capsules (Gong and Jiang, 2015; Zhang et al., 2015; Chang et al., 2017), and one study combined paroxetine capsules (Xiao, 2021) in two groups. TCM treatments included Xiaoyao powder/pill (Zhang et al., 2015; Sun et al., 2016; Wang et al., 2019; Jin et al., 2020; Xiao, 2021), Shugan Xiaopi formula (31), Shugan Xiaobi prescription (Chang et al., 2017), Kuntai capsule (Liu et al., 2020), Gan-Mai Da-Zao decoction, and Yueju pill (Chen, 2017). The composition and source of the formulations are described in detail in Supplementary Material S3.

**TABLE 1:** Study characteristics of included studies.

Study ID	Sample size (I/C)	Age (year)		Disease course		Surgery type (I/C)	Pathological type (I/C)	Stage (I/C)	Clinical intervention		Intervention time (week)	Outcome
		I	C	I	C				I	C		
Chang (2017)	42/42	49.30 ± 4.00	48.6 ± 3.20	N	N		Infiltrating ductal carcinoma (35/36); intraductal carcinoma (4/3); medullary carcinoma (2/1); carcinoma simplex (1/2)	Ⅱ (26/28); Ⅲa-Ⅲb (12/12); Ⅲc (4/2)	Shugan Xiaobi prescription + fluoxetine and CMF chemotherapy	Fluoxetine and CMF chemotherapy	4	① ③
Chen (2017)	35/35	47.23 ± 7.59	46.18 ± 2.26	N	N	All were modified radical operation	All were infiltrating ductal carcinoma	All were Ⅱ-Ⅲ	Gan-Mai Da-Zao decoction and Yueju decoction + psychological counseling	Psychological counseling	2	②
Gong (2015)	50/40	49.4 ± 12.3	50.1 ± 11.7	N	N	Modified radical operation (35/30); standard radical operation (13/9); breast conserving surgery (2/1)			Shugan Xiaopi formula + symptomatic and supportive treatment and fluoxetine capsules	Symptomatic and supportive treatment, and fluoxetine capsules	8	① ② ③
Jin (2020)	50/50	50.56 ± 2.49	51.53 ± 8.64	5.67 ± 2.87 (M)	5.64 ± 2.84 (M)				Modified Xiaoyao powder + conventional chemotherapy, and psychological counseling	Conventional chemotherapy and psychological counseling	6	① ②
Liu (2020)	44/43	53.19 ± 5.09	53.14 ± 5.02	2.01 ± 0.18 (Y)	1.99 ± 0.21 (Y)			Ⅰ (9/10); Ⅱ (22/21); Ⅲ (13/12)	Kuntai capsule + TE chemotherapy and psychological counseling	TE chemotherapy and psychological counseling	6	① ③ ④ ⑤ ⑥ ⑦ ⑧ ⑨ ⑩
Sun (2016)	32/32	45.9 ± 11.7	46.4 ± 12.3	1–14 (M)	1–14 (M)				Modified Xiaoyao powder + TE chemotherapy and psychological counseling)	TE chemotherapy and psychological counseling	6	② ④ ⑤ ⑥
Wang (2019)	67/67	42.3 ± 12.34	40.57 ± 13.91	4.38 ± 3.77 (M)	5.12 ± 4.19 (M)	Modified radical operation (62/61); breast conserving surgery (5/6); postoperative recurrence (8/9)			Xiaoyao powder + postoperative conventional anti-infection, nutritional support, and symptomatic therapy	Postoperative conventional anti-infection, nutritional support, and symptomatic therapy	4	① ⑦ ⑧ ⑨ ⑩
Xiao (2021)	50/50	45.81 ± 11.56	46.37 ± 12.2	N	N		Infiltrating ductal carcinoma (21/23); invasive lobular carcinoma (22/21); other (7/6)	Ⅰ (12/11); Ⅱ (25/24); Ⅲ (9/12); Ⅳ (4/3)	Modified Xiaoyao powder + paroxetine hydrochloride tablets	Paroxetine hydrochloride tablets	12	① ③ ④ ⑤ ⑥
Zhang (2015)	30/30	49.2	46.8	1.0–4.5 (Y)	1.5–5.7 (Y)			Ⅰ-Ⅲ: 41; Ⅳ: 19	Modified Xiaoyao powder + fluoxetine hydrochloride capsules	Fluoxetine hydrochloride capsules	3	① ③

I, intervention group; C, control group; N, none; Y, year; M, month; TE, epirubicin and docetaxel chemotherapy; CMF, cyclophosphamide monohydrate, methotrexate, and fluorouracil; Disease course: the time since diagnosis. ①HAMD; ② SDS; ③ clinical efficacy; ④ 5-HT; ⑤ DA; ⑥ NE; ⑦ CD3; ⑧ CD4; ⑨ CD8; ⑩ CD4/CD8.

### Methodological quality of included studies

**FIGURE 2:**
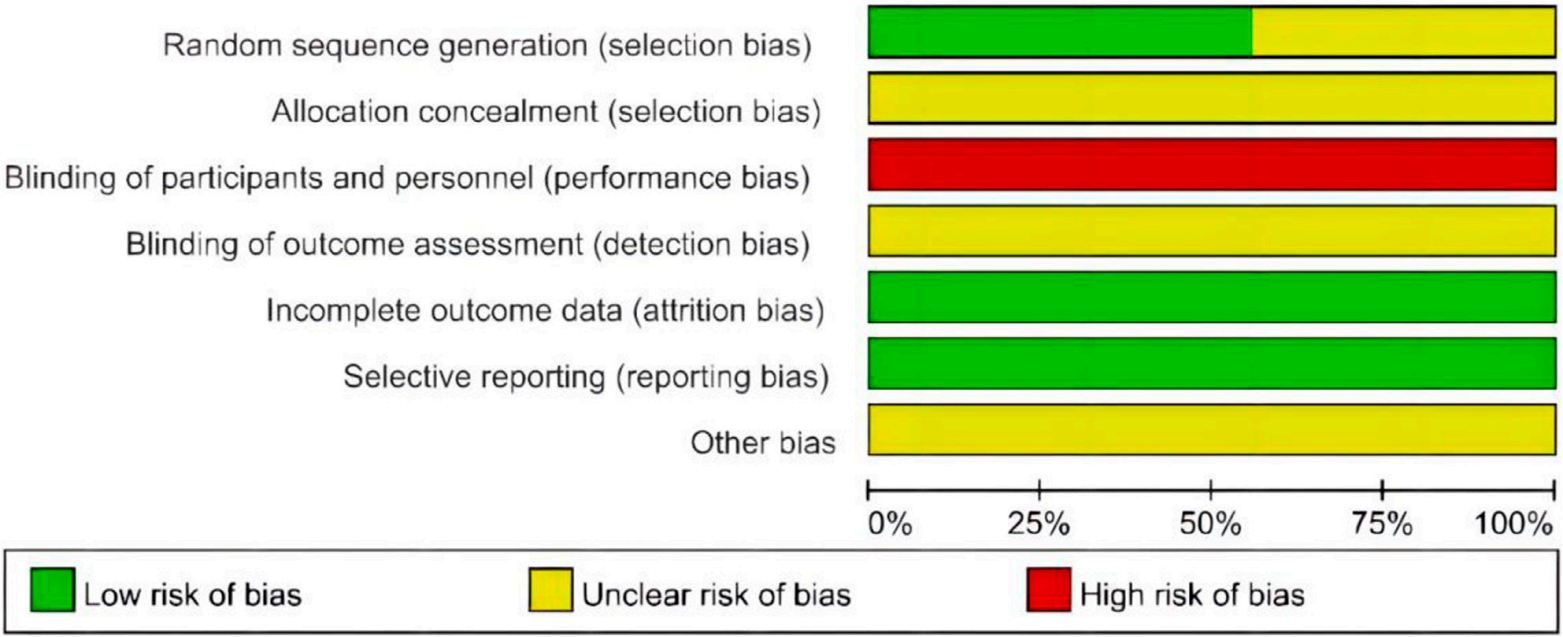
Quality assessment of the included trials risk of bias graph.

Five RCTs used the random number tables method and were assessed as low risk of random sequence generation (Sun et al., 2016; Chang et al., 2017; Wang et al., 2019; Liu et al., 2020; Xiao, 2021), and the other four RCTs did not elaborate on specific methods of randomization, and the risks were unclear (Gong and Jiang, 2015; Zhang et al., 2015; Chen, 2017; Jin et al., 2020). None of the included studies explicitly mentioned the use of allocation concealment and the blind method, which led to a high or unclear risk of bias in the relative domain. None of the nine studies had data missing or missing data that were comparable in each intervention group, and the reasons for missing data were similar, so they were rated as having a low risk of attrition bias. Nine studies were all at low risk of reporting bias (Figure 2). The GRADE method to rate the quality of evidence across studies was applied, and the quality of HAMD, clinical efficacy, and CD3^+^ was moderate. The quality of evidence of included studies is shown in Table 2.

**TABLE 2:** Quality of evidence of included studies.

Question: Should treatment with TCM or without TCM be used for HAMD?												
Quality assessment							No. of patients		Effect		Quality	Importance
No. of studies	Design	Risk of bias	Inconsistency	Indirectness	Imprecision	Other considerations	With TCM	Without TCM	Relative (95% CI)	Absolute		
**1. HAMD (better indicated by lower values)**												
7	Randomized trials	Serious^1^	No serious inconsistency	No serious indirectness	No serious imprecision	None	333	322	-	MD 4.22 lower (5.55–2.88 lower)	⊕⊕⊕Ο MODERATE	
**1.1.1 With chemotherapy (better indicated by lower values)**												
3	Randomized trials	Serious^1^	No serious inconsistency	No serious indirectness	No serious imprecision	None	136	135	-	MD 3.8 lower (5.22–2.38 lower)	⊕⊕⊕Ο MODERATE	
**1.1.2 Without chemotherapy (better indicated by lower values)**												
4	Randomized trials	Serious^1^	No serious inconsistency	No serious indirectness	No serious imprecision	None	197	187	-	MD 4.66 lower (7.02–2.31 lower)	⊕⊕⊕Ο MODERATE	
**1.2.1 Xiaoyao powder/pill (better indicated by lower values)**												
4	Randomized trials	Serious^1^	No serious inconsistency	No serious indirectness	No serious imprecision	None	197	197	-	MD 4.44 lower (6.98–1.9 lower)	⊕⊕⊕Ο MODERATE	
**1.2.2 Other (better indicated by lower values)**												
3	Randomized trials	Serious^1^	No serious inconsistency	No serious indirectness	No serious imprecision	None	136	125	-	MD 4.16 lower (5.55–2.78 lower)	⊕⊕⊕Ο MODERATE	

**Question:** Should with TCM or without TCM be used for clinical efficacy?												
Quality assessment							No. of patients		Effect		Quality	Importance
No of studies	Design	Risk of bias	Inconsistency	Indirectness	Imprecision	Other considerations	Clinical efficacy	Control	Relative (95% CI)	Absolute		
**Clinical efficacy (better indicated by lower values)**												
5	Randomized trials	Serious^1^	No serious inconsistency	No serious indirectness	No serious imprecision	None	195/216 (90.3%)	144/205 (70.2%)	RR 1.25 (1.14–1.37)	176 more per 1,000 (from 98 more to 260 more)	⊕⊕⊕Ο MODERATE	
								69.1%	173 more+. per 1,000 (from 97 more to 256 more)			

**Question:** Should treatment with TCM or without TCM be used for CD3^+^?												
Quality assessment							No. of patients		Effect		Quality	Importance
No. of studies	Design	Risk of bias	Inconsistency	Indirectness	Imprecision	Other considerations	CD3	Control	Relative (95% CI)	Absolute		
**CD3^+^ (better indicated by lower values)**												
2	Randomized trials	Serious^1^	No serious inconsistency	No serious indirectness	No serious imprecision	None	111	110	-	MD 15.18 higher (13.61–16.75 higher)	⊕⊕⊕Ο MODERATE	

### Depression status

#### HAMD

**FIGURE 3:**
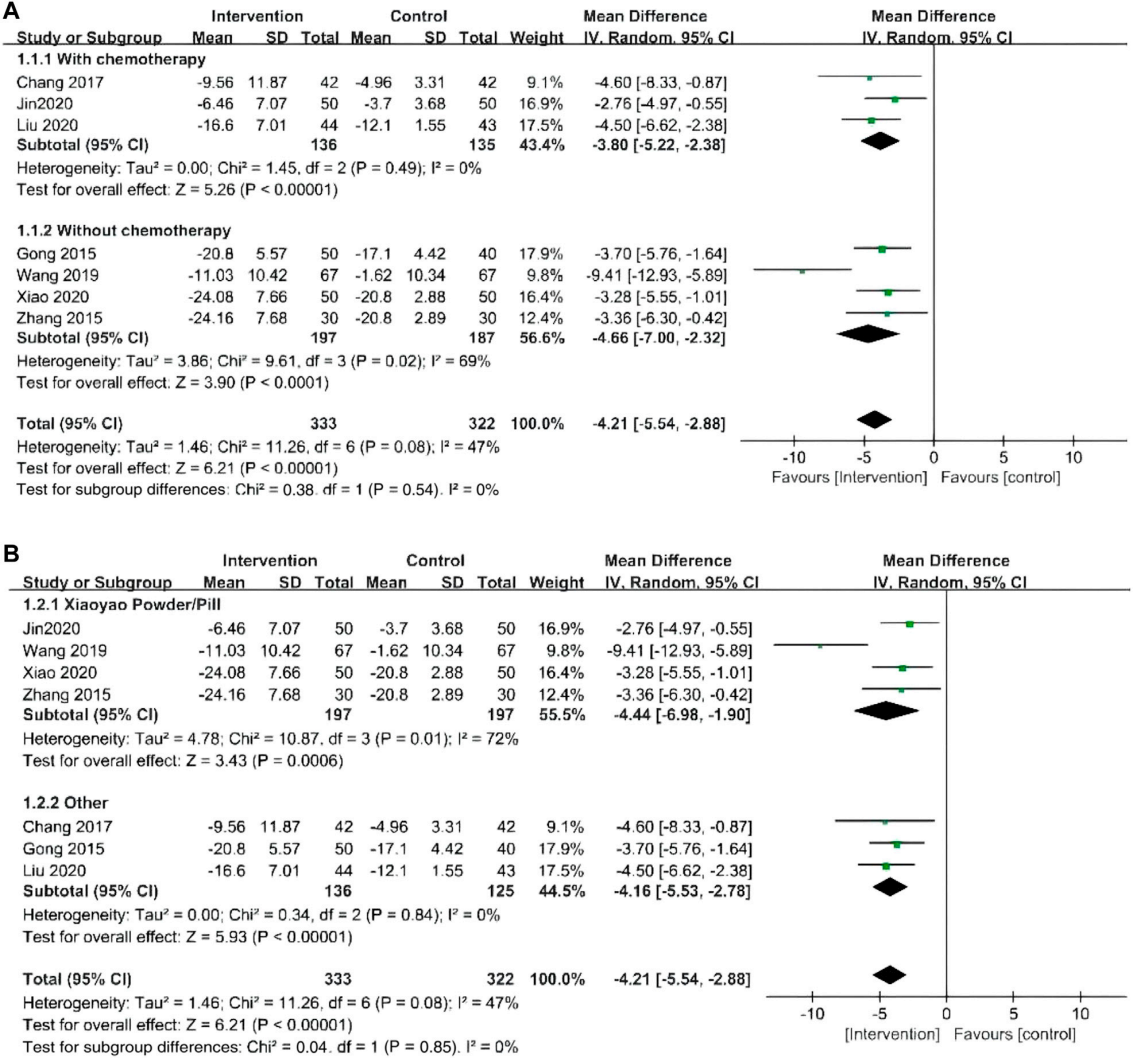
**(A)** Forest plots of HAMD by whether combined chemotherapy or not; **(B)** Forest plots of HAMD by whether to use Xiaoyao Powder/Pill or other traditional Chinese medicines.

A total of seven RCTs with 655 patients evaluated depression status with HAMD (Gong and Jiang, 2015; Zhang et al., 2015; Chang et al., 2017; Wang et al., 2019; Jin et al., 2020; Liu et al., 2020; Xiao, 2021). The meta-analysis showed that the effect of the intervention group was significantly better than that of the control group in reducing the HAMD score (MD = −4.21, 95% CI −5.54 to −2.88). Further subgroup analysis was conducted according to whether combined chemotherapy was used or not and whether Xiaoyao powder/pill or other traditional Chinese medicines were used. Subgroup analysis showed no statistical difference in whether chemotherapy (*p* = 0.54) and Xiaoyao Powder were used. (*p* = 0.85) In the sensitivity analysis, the results showed that one study was the main source of heterogeneity, which may be related to the fact that this RCT (Wang et al., 2019) only received postoperative anti-infective therapy and did not receive psychological treatment (including antidepressant therapy or psychological counseling). The forest plot is shown in Figure 3.

#### SDS

**FIGURE 4:**
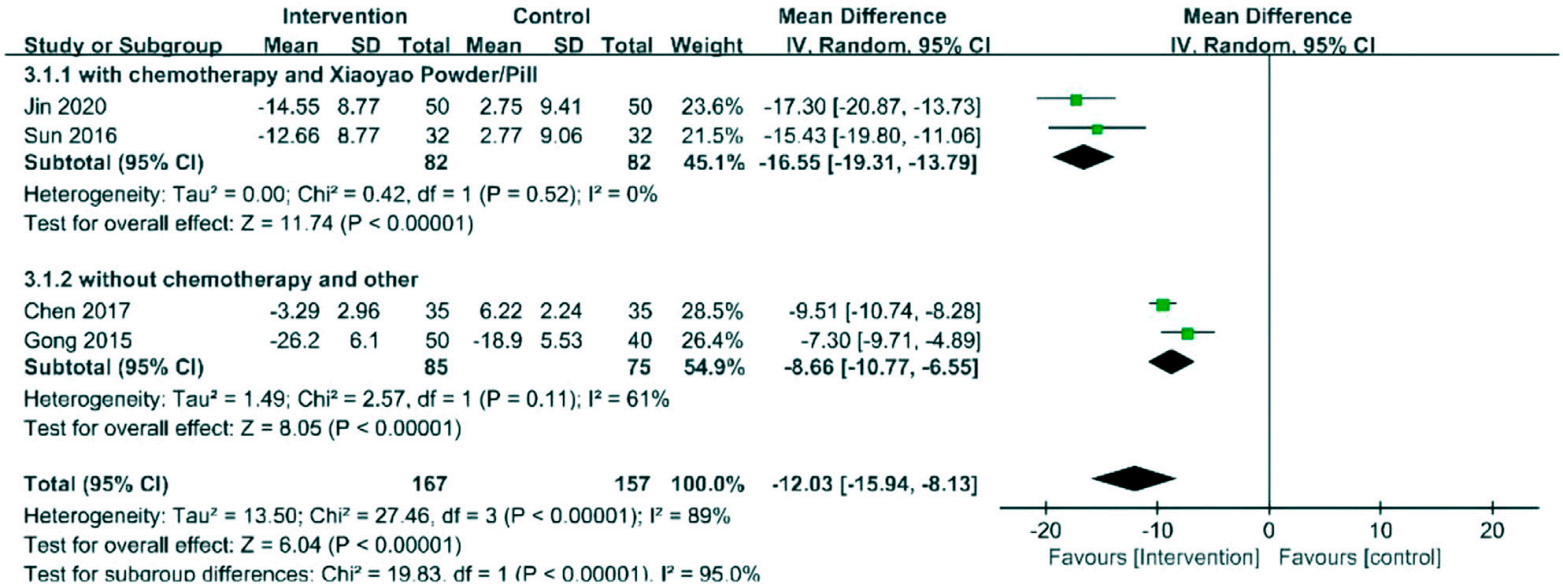
Forest plots of SDS.

Four RCTs with 324 patients evaluated depression status with SDS (Gong and Jiang, 2015; Chen, 2017; Jin et al., 2020; Liu et al., 2020). The results showed that the intervention group had a more significant effect on decreasing the SDS score (MD = −12.03, 95% CI −15.94 to −8.13). Xiaoyao powder/pill was used in two studies that combined chemotherapy, and other traditional Chinese medicines were used in two studies without combined chemotherapy. Subgroup analysis was conducted according to whether chemotherapy was received and whether Xiaoyao powder/pill or other medicines were used. The result of the subgroup analysis suggested that the SDS score of patients treated with Xiaoyao powder/pill at the chemotherapy stage decreased more significantly than that of those treated with other traditional Chinese medicines at the non-chemotherapy stage (*p* < 0.00001). The forest plot is shown in Figure 4.

### Clinical efficacy

**FIGURE 5:**
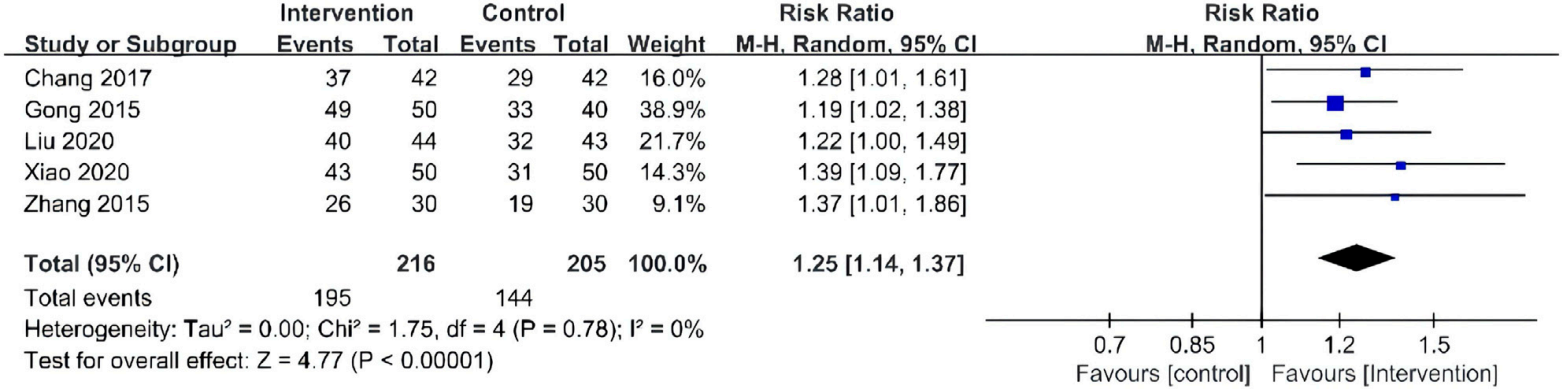
Forest plots of clinical efficacy.

Five RCTs reported clinical efficacy in outcome indicators (Gong and Jiang, 2015; Zhang et al., 2015; Chang et al., 2017; Liu et al., 2020; Xiao, 2021). The results showed that compared to the patients who received conventional therapies, those who received TCM plus conventional therapies have a significantly better clinical efficacy (RR = 1.25, 95% CI 1.14–1.37). The forest plot is shown in Figure 5.

### Levels of laboratory tests

#### 5-HT, DA, and NE

**FIGURE 6:**
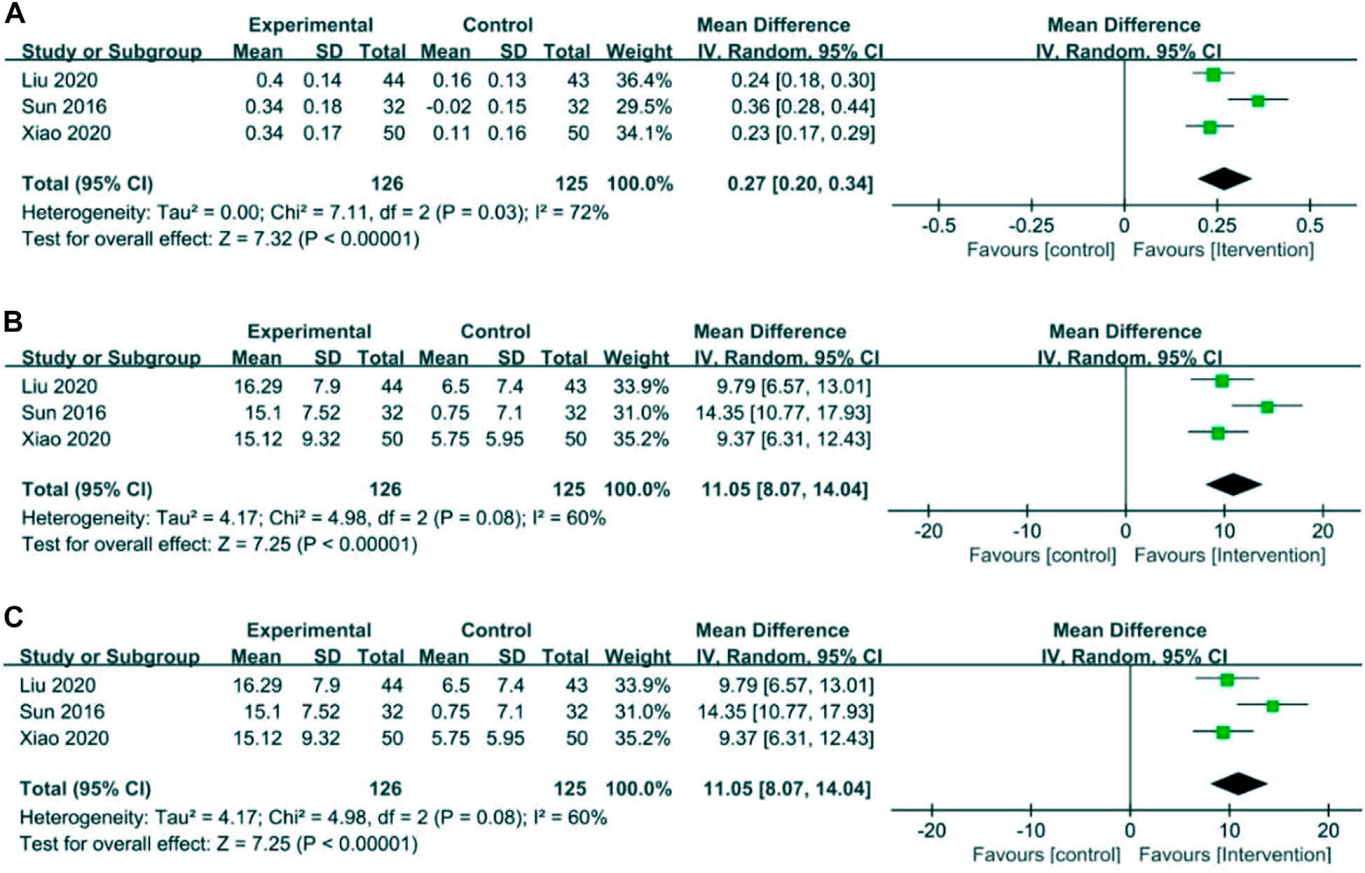
Forest plots of 5-HT **(A)**, DA **(B)**, and NE **(C)**.

Three RCTs involving 126 cases in the intervention group and 125 cases in the control group reported 5-HT, DA, and NE in the outcome indicators (Sun et al., 2016; Xiao, 2021). Meta-analysis showed that compared to control groups, intervention groups were significantly better in increasing 5-HT (MD = 0.27, 95% CI 0.20–0.34), DA (MD = 26.48, 95% CI 24.18–28.77), and NE (MD = 11.05, 95% CI 8.07–14.04). The results are presented in Figure 6.

### Immune function index

#### CD3^+^, CD4^+^, CD8^+^, and CD4^+^/CD8^+^

**FIGURE 7:**
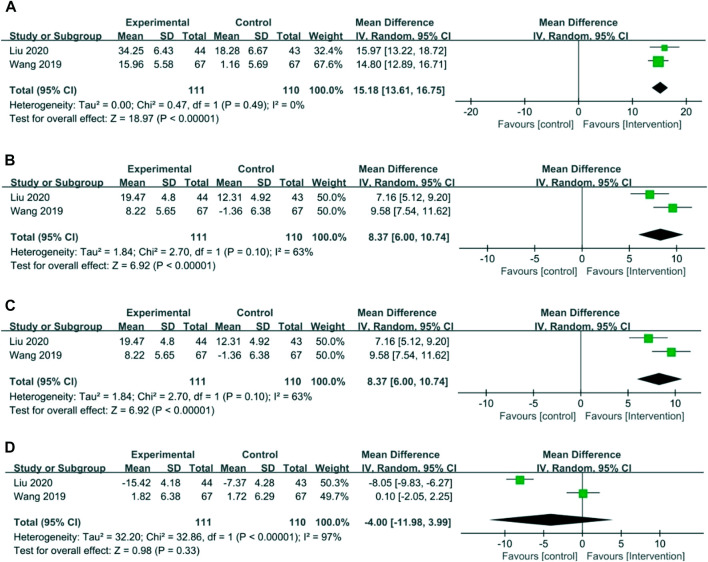
Forest plots of the immune function index. **(A)** CD3^+^, **(B)** CD4^+^, **(C)** CD4^+^/CD8^+^, and **(D)** CD8^+^.

Two RCTs with 221 patients reported an immune function index (Wang et al., 2019; Liu et al., 2020). Meta-analysis showed that the intervention group was greatly improved compared with the control group in increasing CD3^+^ (MD = 15.18, 95% CI 13.61–16.75), CD4^+^ (MD = 8.37, 95% CI 6.00–10.74), and CD4^+^/CD8^+^ (MD = 0.33, 95% CI 0.27–0.39). The results showed that there was no statistical difference between the intervention group and the control group in CD8^+^ (MD = −4.00, 95% CI −11.98 to 3.99). The results are presented in Figure 7.

### Safety outcomes

A study showed that no adverse events, side effects, or TCM treatment-related toxic side effects were reported in the intervention group in the nine RCTs (Wang et al., 2019). Some RCTs showed that the incidence of adverse reactions such as nausea and vomiting, cardiotoxicity, liver and kidney function injury, bone marrow suppression, and alopecia in the intervention group was lower than that in the control group (Sun et al., 2016; Jin et al., 2020; Liu et al., 2020; Xiao, 2021). This indicates that TCM was safe for the treatment of postoperative depression of BC.

### Publication bias

**FIGURE 8:**
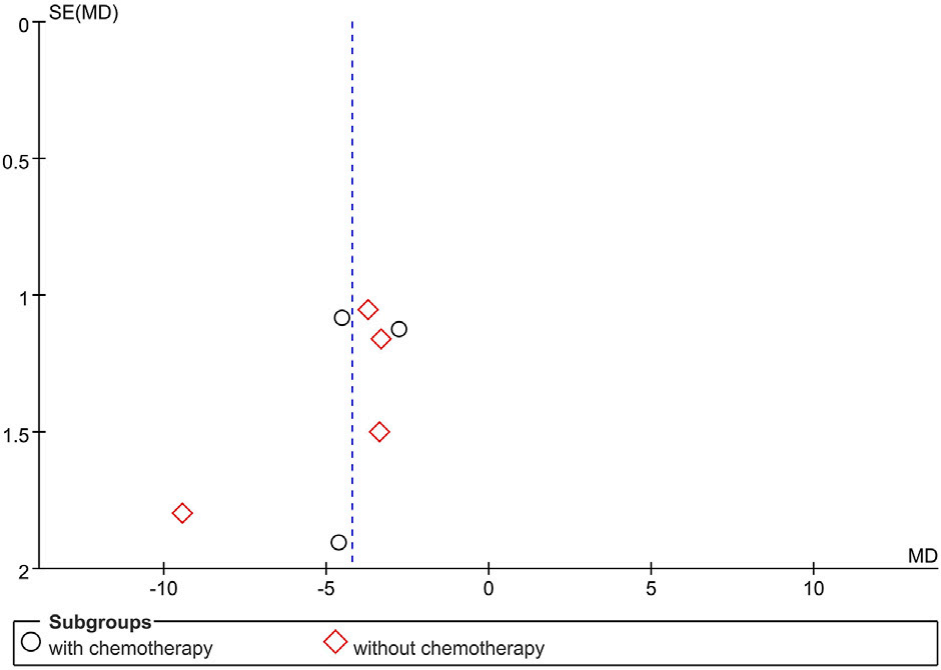
Funnel plot of the trials; HAMD.

The funnel plot of the primary outcome (HAMD) showed no complete symmetry and indicated the existence of publication bias. The result is presented in Figure 8. Publication bias may be associated with negative results not being published.

## Discussion

In this systematic review and meta-analysis of nine RCTs in the postoperative depression of BC, we have used the GRADE method to rate the quality of evidence across studies and have moderate certainty that application of TCM may lead to a better effect on improving the HAMD score. At the same time, the SDS score is reduced to some extent. It also increased the levels of 5-HT, DA, and NE in the serum and improved the immune index of CD3^+^, CD4^+^, and CD4^+^/CD8^+^.

The immune ability of the patients directly affects the development of tumors, and T lymphocytes play an indispensable role in the fight against tumors. CD4^+^ T lymphocytes can activate CD8^+^ T lymphocytes through a variety of pathways to kill tumor cells, and CD3^+^-induced lymphocyte proliferation plays a central role in the immune response cascade (McGregor et al., 2004; Yuan et al., 2023). One study showed that higher levels of CD3^+^, CD4^+^, and CD8^+^ were associated with longer survival (Castaneda et al., 2016).

The literature on the immune index and depression appears inconclusive. Most studies have indicated that CD3^+^ and CD4^+^ are negatively correlated with depression, and the increased CD3^+^ and CD4^+^ levels may improve the depression status of patients (Laumet et al., 2020; Lu et al., 2021). However, some studies have shown that the higher levels of CD4^+^ and CD8^+^ among active treatment BC patients may cause the poorer quality of life and sleep quality (Boivin et al., 2020). There was considerable controversy regarding the relationship between CD8^+^ and depression. Some studies have indicated that a higher CD8^+^ and CD4^+^/CD8^+^ ratio in people with depression may lead to worse depressive symptoms (Zorrilla et al., 2001; Boivin et al., 2020). However, another research has shown that the CD4^+^/CD8^+^ ratio was negatively associated with depression (Laumet et al., 2020). The relationship between the immune index and depression may weaken after treatment and may relate to the type of depression (Yang Shengliang et al., 2021; Lu et al., 2021). Although the relationship between the CD^+^ marker and depression is still controversial, it can be concluded in this meta-analysis that TCM can significantly reduce the depression status of patients. Current antidepressants that are used in Western medicine usually have a single pathway of action, significant side effects, and modest curative effects. However, TCM has the characteristics of multiple targets, high safety, and good clinical efficacy, which may offer new options for the treatment of cancer-related depression (Lin et al., 2017).

In this meta-analysis, there was no significant difference in HAMD scores between the two groups reported by two RCTs, but significant differences were observed in SDS scores in the same RCTs (Gong and Jiang, 2015; Jin et al., 2020). There are possible reasons for this result: HAMD score is a professional tool for doctors to assess the degree of depression and objectively evaluate the overall condition of patients through other rating methods. In the SDS score, patients were scored according to the frequency and degree of their symptoms to avoid evaluation errors caused by unclear expression, and the score can reflect the severity of symptoms. Some of the negative feelings were also evident in normal people in times of adversity, which may lead to SDS scores higher than in the normal population. Both of them could reflect the severity of depression from subjective and objective perspectives, respectively. HAMD is more accurate than SDS in distinguishing the severity of depression. Some studies showed there were significant differences in SDS and HAMD scores between different severity depression groups, and the consistency between the severity of symptoms derived from SDS and the severity level derived from the existing severity classification criteria is not high. The evaluation value of the HAMD score is generally higher than that of SDS (Liu Baoyan et al., 2021). Another study showed that the SDS grade was not consistent with the severity of symptoms obtained by HAMD. The sensitivity and specificity of SDS were not satisfactory. Therefore, the depression scale should be used rationally in clinical practice to enhance the accuracy of evaluation (Duan and Sheng, 2012).

It is believed that the pathogenesis of depression is related to the hypothalamic–pituitary–adrenal (HPA) axis, the gut–brain–microbiome axis, brain-derived neurotrophic factor (BDNF), monoamine neurotransmitters, and cytokines (Kubera et al., 2011; Dooley et al., 2016; Zhao J. et al., 2022; Flores et al., 2022; Maybee et al., 2022). Studies have shown that Gan-Mai Da-Zao decoction can increase the content of monoamine neurotransmitters in patients with depression, regulate the function of the monoamine system, reduce central and peripheral pro-inflammatory factors, and inhibit HPA axis hyperactivity. It can increase the mRNA and protein expression of BDNF, thereby exerting the clinical effect of antidepressants (Yang Xuejing et al., 2021). Xiaoyao power can also improve gastrointestinal function and depression by affecting the gut–brain–microbiome axis and the neuro–endocrine–immune axis (RenQi et al., 2021; Zhao D. et al., 2022). The present study suggests that albiflorin and paeoniflorin may treat cancer-related depression by modulating the neuroendocrine-immune networks and aberrant metabolic pathways (Wang et al., 2021). TCM may increase the content of 5-HT, DA, and NE in blood and affect immunity through the aforementioned ways to play an anti-depressant effect.

At present, there are few relevant clinical studies and a lack of data on the treatment of depression after BC surgery with TCM, almost no double-blinded studies, and the literature quality is poor. More high-quality RCTs should be conducted in the future. Most of the nine RCTs in this meta-analysis were treated with TCM combined with chemotherapy. More RCTs can be conducted on postoperative depression of BC after chemoradiotherapy or without chemoradiotherapy to avoid distractions from other treatments. Most of the original studies included in this meta-analysis did not mention the surgical method and clinical stage of the included patients, although some studies showed that there was no significant relationship between the surgical method and clinical stage in the postoperative depression of BC (Xu et al., 2015) which needed more studies to verify this conclusion in the future.

Studies have shown that psychosocial factors are closely related to the incidence of BC-related depression. Depression is one of the adverse factors in the recurrence and unfavorable evolution of BC (Wang et al., 2020; Schenker et al., 2022; Xu et al., 2022). The incidence of postoperative depression in BC is closely related to physiology, society, and psychology. The contemporary medical model has gradually changed to the bio–psycho–social medical model. TCM maintains physical and mental health through the combination of psychological intervention, emotional adjustment, and TCM treatment, showing the advantages of TCM clinical treatment and providing patients with more reasonable and efficient treatment plans (Liu Xue et al., 2021). However, most of the clinical studies on the treatment of postoperative depression of BC have no quality control and chemical report in the treatments. This is a shortcoming of this article and a limitation of current common assessment tools in TCM. TCM plays an increasingly important role in treating postoperative depression of BC. Therefore, it is necessary to increase further the exploration and conduct more high-quality research on depression after BC surgery with TCM to better apply it in clinical practice.

## Conclusion

Overall, TCM can be an add-on therapy for postoperative depression of BC for improving symptoms of depression, increasing the 5-HT, DA, and NE levels in the blood, and raising the immune index of patients with a significant and safe effect. More randomized clinical trials and extended follow-ups are needed to evaluate the effect of TCM on postoperative depression of BC.

## Data Availability

The original contributions presented in the study are included in the article/Supplementary Material; further inquiries can be directed to the corresponding author.
