# Supplementary material for: Effect of traditional Chinese medicine on postoperative depression of breast cancer: a systematic review and meta-analysis
Source: Front Pharmacol. 2023 Jun 23;14:1019049. doi: 10.3389/fphar.2023.1019049 (PMC10327430; doi:10.3389/fphar.2023.1019049)
Supplement: Supplementary file 1 [file DataSheet1.doc]

Supplementary File 1. PRISMA-2009 Checklist

| **Section/topic** | **#** | **Checklist item** | **Reported on page #** |
| --- | --- | --- | --- |
| **TITLE** | | |  |
| Title | 1 | Identify the report as a systematic review, meta-analysis, or both. | Page 1 |
| **ABSTRACT** | | |  |
| Structured summary | 2 | Provide a structured summary including, as applicable: background; objectives; data sources; study eligibility criteria, participants, and interventions; study appraisal and synthesis methods; results; limitations; conclusions and implications of key findings; systematic review registration number. | Page 1-2 |
| **INTRODUCTION** | | |  |
| Rationale | 3 | Describe the rationale for the review in the context of what is already known. | Page 2-3 |
| Objectives | 4 | Provide an explicit statement of questions being addressed with reference to participants, interventions, comparisons, outcomes, and study design (PICOS). | Page 4 |
| **METHODS** | | |  |
| Protocol and registration | 5 | Indicate if a review protocol exists, if and where it can be accessed (e.g., Web address), and, if available, provide registration information including registration number. | None |
| Eligibility criteria | 6 | Specify study characteristics (e.g., PICOS, length of follow-up) and report characteristics (e.g., years considered, language, publication status) used as criteria for eligibility, giving rationale. | Page 5 |
| Information sources | 7 | Describe all information sources (e.g., databases with dates of coverage, contact with study authors to identify additional studies) in the search and date last searched. | Page 4 |
| Search | 8 | Present full electronic search strategy for at least one database, including any limits used, such that it could be repeated. | Supplementary File 2 |
| Study selection | 9 | State the process for selecting studies (i.e., screening, eligibility, included in systematic review, and, if applicable, included in the meta-analysis). | Page 6 |
| Data collection process | 10 | Describe method of data extraction from reports (e.g., piloted forms, independently, in duplicate) and any processes for obtaining and confirming data from investigators. | Page 6 |
| Data items | 11 | List and define all variables for which data were sought (e.g., PICOS, funding sources) and any assumptions and simplifications made. | Page 6 |
| Risk of bias in individual studies | 12 | Describe methods used for assessing risk of bias of individual studies (including specification of whether this was done at the study or outcome level), and how this information is to be used in any data synthesis. | Page 7 |
| Summary measures | 13 | State the principal summary measures (e.g., risk ratio, difference in means). | Page 7 |
| Synthesis of results | 14 | Describe the methods of handling data and combining results of studies, if done, including measures of consistency (e.g., I^2^) for each meta-analysis. | Page 7 |
| Risk of bias across studies | 15 | Specify any assessment of risk of bias that may affect the cumulative evidence (e.g., publication bias, selective reporting within studies). | Page 9 |
| Additional analyses | 16 | Describe methods of additional analyses (e.g., sensitivity or subgroup analyses, meta-regression), if done, indicating which were pre-specified. | Page 7 |
| **RESULTS** | | |  |
| Study selection | 17 | Give numbers of studies screened, assessed for eligibility, and included in the review, with reasons for exclusions at each stage, ideally with a flow diagram. | Page 8 Figure 1 |
| Study characteristics | 18 | For each study, present characteristics for which data were extracted (e.g., study size, PICOS, follow-up period) and provide the citations. | Page 11-12  Table 1 |
| Risk of bias within studies | 19 | Present data on risk of bias of each study and, if available, any outcome level assessment (see item 12). | Page 10  Figure 2 |
| Results of individual studies | 20 | For all outcomes considered (benefits or harms), present, for each study: (a) simple summary data for each intervention group (b) effect estimates and confidence intervals, ideally with a forest plot. | Page 10-15  Figure 3-7 |
| Synthesis of results | 21 | Present results of each meta-analysis done, including confidence intervals and measures of consistency. | Page 10-15  Figure 3-7 |
| Risk of bias across studies | 22 | Present results of any assessment of risk of bias across studies (see Item 15). | Page 16  Figure 8 |
| Additional analysis | 23 | Give results of additional analyses, if done (e.g., sensitivity or subgroup analyses, meta-regression [see Item 16]). | Page 16 |
| **DISCUSSION** | | |  |
| Summary of evidence | 24 | Summarize the main findings including the strength of evidence for each main outcome; consider their relevance to key groups (e.g., healthcare providers, users, and policy makers). | Page 16-17 |
| Limitations | 25 | Discuss limitations at study and outcome level (e.g., risk of bias), and at review-level (e.g., incomplete retrieval of identified research, reporting bias). | Page 17-18 |
| Conclusions | 26 | Provide a general interpretation of the results in the context of other evidence, and implications for future research. | Page 20 |
| **FUNDING** | | |  |
| Funding | 27 | Describe sources of funding for the systematic review and other support (e.g., supply of data); role of funders for the systematic review. | Page 21 |

Supplementary File 2. Search strategy used in PubMed

#1 (Medicine, Chinese Traditional[MeSH Terms]) OR (Botanical Drugs[MeSH Terms])

#3 ((((((((((((((Botanical Drugs Medicine[Title/Abstract]) OR (Medicine, Botanical Drugs[Title/Abstract])) OR (Medicine, Chinese Traditional[Title/Abstract])) OR (Traditional Chinese Medicine[Title/Abstract])) OR (Traditional Medicine, Chinese[Title/Abstract])) OR (Chinese Traditional Medicine[Title/Abstract])) OR (Chinese Medicine, Traditional[Title/Abstract])) OR (Drugs, Chinese Botanical Drugs[Title/Abstract])) OR (Chinese Drugs, Plant[Title/Abstract])) OR (Chinese Botanical Drugs[Title/Abstract])) OR (Botanical Drugs, Chinese[Title/Abstract])) OR (Plant Extracts, Chinese[Title/Abstract])) OR (Chinese Plant Extracts[Title/Abstract])) OR (Extracts, Chinese Plant[Title/Abstract])) OR ([Combination Chinese Traditional and Western Medicine](javascript:;)[Title/Abstract])

#3 #1 or #2

#4 Breast Neoplasms[MeSH Terms]

#5 (((((((((((((((((Neoplasm, Breast[Title/Abstract]) OR (Breast Neoplasm[Title/Abstract])) OR (Neoplasms, Breast[Title/Abstract])) OR (Mammary Neoplasms[Title/Abstract])) OR (Mammary Neoplasm[Title/Abstract])) OR (Neoplasm, Mammary[Title/Abstract])) OR (Neoplasms, Mammary[Title/Abstract])) OR (Cancer of Breast[Title/Abstract])) OR (Breast Cancers[Title/Abstract])) OR (Mammary Cancer[Title/Abstract])) OR (Cancer, Mammary[Title/Abstract])) OR (Cancers, Mammary[Title/Abstract])) OR (Mammary Cancers[Title/Abstract])) OR (Mammary Carcinoma[Title/Abstract])) OR (Breast Cancer[Title/Abstract])) OR (Cancer, Breast[Title/Abstract])) OR (Cancers, Breast[Title/Abstract])) OR (Cancer of the Breast[Title/Abstract])

#6 #4 OR #5

#7 Postoperative Period[MeSH Terms]

### #8 ((((((Postoperative[Title/Abstract]) OR (Post Operation[Title/Abstract])) OR (Postoperative Period[Title/Abstract])) OR (After Operation[Title/Abstract])) OR (After Surgery[Title/Abstract])) OR (Post Operative[Title/Abstract])) OR (Postoperatiue Period[Title/Abstract])

#9 #7 OR #9

#10 (Depression[MeSH Terms]) OR (Depressive Disorder[MeSH Terms])

#11 (((((Depression[Title/Abstract]) OR (Depressive Disorder[Title/Abstract])) OR (Depressive[Title/Abstract])) OR (Mood Disorder[Title/Abstract])) OR (Negative Emotion[Title/Abstract])) OR (Negative Mood[Title/Abstract])

#12 #10 OR #11

#13 ((randomized controlled trial[pt] OR controlled clinical trial[pt] OR randomized[tiab] OR placebo[tiab] OR clinical trials as topic[mesh:noexp] OR randomly[tiab] OR trial[ti] NOT (animals[mh] NOT humans [mh])))

#14 #3 AND #6 AND #9 AND #12 AND #13

Supplementary File 3. Summary of all of the included trails

| Study | Formulation | Source | Species, concentration | Quality control reported? (Y/N) | Chemical analysis reported?  (Y/N) |
| --- | --- | --- | --- | --- | --- |
| Chang 2017 | Shugan Xiaobi Prescription | Liquan County People's Hospital, Xianyang City, Shaanxi Province | ·Paeonia lactiflora Pall. [Paeoniaceae; Paeoniae Radix Alba.]  30g  ·Ostrea gigas Thunberg. [[Ostreidae](javascript:;); Ostreae Concha.] 30g  ·Lonicera japonica Thunb. [Caprifoliaceae; Lonicerae Japonicae Flos.] 20g  ·Semiaquilegia adoxoides (DC.) Makino. [Ranunculaceae; Muskroot-like-like Semiaquilegia Root.] 20g  ·Paeonia lactiflora Pall. [Paeoniaceae; Paeoniae Radix Alba.] 15g  ·Pinellia ternata (Thunb.) Makino.[Araceae Juss; Pinelliae Rhizoma..] 15g  ·Citrus reticulata Blanco. [Rutaceae; Citri Reticulatae Pericarpium..] 15g  ·Curcuma wenyujin Y. H. Chen et C. Ling. [Zingiberaceae; Curcumae Radix.] 15g  ·Citrus × aurantium L. [Rutaceae; Fructus Aurantii Immaturus.] 15g  ·Bupleurum chinense DC. [Apiaceae Lindl.; Bupleuri Radix.] 15g  ·Poria cocos (Schw.) Wolf. [Polyporaceae; Poria.] 15g  ·Sargassum. [Sargassaceae; Sargassum pallidum (Turn.) C. Ag. or Sargassum fusiforme (Harv.) Setch.] 10g  ·Glycyrrhiza uralensis Fisch. [Fabaceae Lindl.; Glycyrrhizae Radix et Rhizoma Praeparata Cum Melle.] 10g  ·Boswellia carterii Birdw. [Burseraceae Kunth; Olibanum.] 10g  ·Commiphora myrrha Engl. [Burseraceae Kunth; Myrrha.] 10g | N | N |
| Chen 2017 | Ganmai Dazao Decoction and Yueju Decoction | - | ·Glycyrrhiza uralensis Fisch. [Fabaceae Lindl.; Glycyrrhizae Radix et Rhizoma Praeparata Cum Melle.] 9g  ·Triticum aestivum. [Poaceae Barnhart; Fructus Triticum aestivumL.] 30g  ·Ziziphus jujuba Mill. [Rhamnaceae Juss.; Jujubae Fructus.] 10pieces  ·Cyperus rotundus L. [Cyperaceae Juss.; Cyperi Rhizoma.] 6g  ·Ligusticum chuanxiong Hort. [Apiaceae Lindl.; Chuanxiong Rhizoma.] 6g  ·Gardenia jasminoides Ellis. [Rubiaceae Juss.; Fructus Gardeniae Preparatus.] 6g  ·Atractylodes lancea (Thunb.) DC. [Asteraceae Bercht. & J. Presl; Atractylodis Rhizoma.] 10g  ·Medicated Leaven.. [-; Massa Medicata Fermentata.] 15g | N | N |
| Gong 2015 | Shugan Xiaopi Formula | ， - | ·Bupleurum chinense DC. [Apiaceae Lindl.; Bupleuri Radix.] 10g  ·Ganoderma lucidum (Leyss.ex Fr.) Karst. [Polyporaceae; Ganoderma.] 10g  ·Panax quinquefolius L. [Araliaceae Juss.; American Ginseng Radix et Rhizoma.] 10g  ·Sparganium stoloniferum Buch.-Ham. [Sparganiaceae; Sparganii Rhizoma.] 10g  ·Curcuma phaeocaulis Val. [Zingiberaceae; Curcumae Rhizoma.] 10g  ·Gypsophila vaccaria (L.) Sm. [Caryophyllaceae Juss.; Fructus Vaccaria segetalis( Neck.)Garcke.] 10g  ·Sargassum. [Sargassaceae; Sargassum pallidum (Turn.) C. Ag. or Sargassum fusiforme (Harv.) Setch.] 10g  ·Ziziphus jujuba Mill. var. spinosa (Bunge) Hu ex H. F. Chou. [Rhamnaceae Juss.; Ziziphi Spinosae Semen.] 10g  ·Cyperus rotundus L. [Cyperaceae Juss.; Cyperi Rhizoma.] 12g  ·Rehmannia glutinosa Libosch. [Scrophulariaceae Juss.; Rehmanniae Radix Praeparata.] 20g  ·Lycium barbarum L. [Solanaceae Juss.; Lycii Fructus.] 20g  ·Citrus reticulata Blanco. [Rutaceae Juss.; Citri Reticulatae Semen.] 20g  ·Pseudocydonia sinensis (Thouin) C. K. Schneid. [Rosaceae Juss.; fructus chaenomelis lagenariae.] 30g  ·Hordeum vulgare L. [Poaceae Barnhart; Hordei Fructus Germinatus.]30g | N | N |
| Jin 2020 | Modeified Xiaoyao Powder | - | ·Bupleurum chinense DC. [Apiaceae Lindl.; Bupleuri Radix.] 9g  ·Angelica sinensis (Oliv.) Diels. [Apiaceae Lindl.; Angelicae Sinensis Radix.] 6g  ·Paeonia lactiflora Pall. [Paeoniaceae; Paeoniae Radix Alba.] 12g  ·oria cocos (Schw.) Wolf.[Polyporaceae;Poria] 15g  ·Zingiber officinale Roscoe. [Zingiberaceae ; Zingiberis Rhizoma Recens.] 3slices  ·Ziziphus jujuba Mill. [Rhamnaceae Juss.; Jujubae Fructus.] 3 pieces  ·Mentha haplocalyx Briq. [Lamiaceae Martinov; Mentha canadensis Linnaeus.] 6g  ·Glycyrrhiza uralensis Fisch. [Fabaceae Lindl.; Glycyrrhizae Radix et Rhizoma Praeparata Cum Melle.] 9g  ·Rehmannia glutinosa Libosch. [Scrophulariaceae Juss.; Rehmanniae Radix Praeparata.] 15g  ·Ligustrum lucidum Ait. [Oleaceae Hoffmanns. & Link; Ligustri Lucidi Fructus.] 15g  ·Yerbadetajo herb. [Asteraceae Bercht. & J. Presl; Herba Ecliptae.] 15g  ·Alisma plantago-aquatica subsp. orientale (Sam.) Sam. [Alismataceae Vent.; Alismatis Rhizoma.] 9g | N | N |
| Liu 2020 | Kuntai Capsule | [SFDA approval number](javascript:;):Z20000083  (Guiyang Xintiandi Pharmaceutical Co., LTD) | ·Rehmannia glutinosa Libosch. [Scrophulariaceae Juss.; Rehmanniae Radix Praeparata.]  ·Coptis chinensis Franch. [Ranunculaceae Juss.; Coptidis Rhizoma.]  ·Paeonia lactiflora Pall.[Paeoniaceae; Paeoniae Radix Alba].  ·Scutellaria baicalensis Georgi. [Lamiaceae Martinov; Scutellariae Radix.]  ·Colla Corii Asini. [Equidae; Asini Corii Colla.]  ·Poria cocos (Schw.) Wolf. [Polyporaceae; Poria.]  0.5g*4 capsules at a time and three times a day | N | N |
| Sun 2016 | Modeified Xiaoyao Powder | - | ·Bupleurum chinense DC. [Apiaceae Lindl.; Bupleuri Radix.] 9g  ·Angelica sinensis (Oliv.) Diels. [Apiaceae Lindl.; Angelicae Sinensis Radix.] 6g  ·Paeonia lactiflora Pall. [Paeoniaceae; Paeoniae Radix Alba.] 12g  ·Atractylodes macrocephala Koidz. [Apiaceae Lindl.; Atractylodis Macrocephalae Rhizoma.] 15g  ·Poria cocos (Schw.) Wolf. [Polyporaceae; Poria.]15g  ·Zingiber officinale Roscoe. [Zingiberaceae; Zingiberis Rhizoma Recens.] 3 slices  ·Ziziphus jujuba Mill. [Rhamnaceae Juss.; Jujubae Fructus.] 3 pieces  ·Mentha haplocalyx Briq. [Lamiaceae Martinov; Mentha canadensis Linnaeus.] 6g  ·Glycyrrhiza uralensis Fisch. [Fabaceae Lindl.; Glycyrrhizae Radix et Rhizoma Praeparata Cum Melle.] 9g  ·Rehmannia glutinosa Libosch. [Scrophulariaceae Juss.; Rehmanniae Radix Praeparata.] 15g  ·Ligustrum lucidum Ait. [Oleaceae Hoffmanns. & Link; Ligustri Lucidi Fructus.] 15g  ·Yerbadetajo herb. [Asteraceae Bercht. & J. Presl; Herba Ecliptae.] 15g  ·Alisma plantago-aquatica subsp. orientale (Sam.) Sam. [Alismataceae Vent.; Alismatis Rhizoma.] 9g | N | N |
| Wang 2019 | Xiaoyao Powder | Chinese Medicine Pharmacy of PLA General Hospital | ·Bupleurum chinense DC. [Apiaceae Lindl.; Bupleuri Radix.] 12g  ·Angelica sinensis (Oliv.) Diels. [Apiaceae Lindl.; Angelicae Sinensis Radix.] 10g  ·Paeonia lactiflora Pall. [Paeoniaceae; Paeoniae Radix Alba.] 10g  ·Poria cocos (Schw.) Wolf. [Polyporaceae; Poria.] 10g  ·Atractylodes macrocephala Koidz. [Apiaceae Lindl.; Atractylodis Macrocephalae Rhizoma.] 10g  ·Mentha haplocalyx Briq. [Lamiaceae Martinov; Mentha canadensis Linnaeus.] 6g  ·Zingiber officinale Roscoe. [Zingiberaceae; Zingiberis Rhizoma Recens.] 6g  ·Glycyrrhiza uralensis Fisch. [Fabaceae Lindl.; Glycyrrhizae Radix et Rhizoma Praeparata Cum Melle.] 6g | N | N |
| Xiao 2021 | Modeified Xiaoyao Powder | - | ·Bupleurum chinense DC. [Apiaceae Lindl.; Bupleuri Radix.] 6g  ·Mentha haplocalyx Briq. [Lamiaceae Martinov; Mentha canadensis Linnaeus.] 6g  ·Glycyrrhiza uralensis Fisch. [Fabaceae Lindl.; Glycyrrhizae Radix et Rhizoma Praeparata Cum Melle.] 6g  ·Codonopsis pilosula (Franch.) Nannf. [Campanulaceae Juss.; Codonopsis Radix.] 15g  ·Poria cocos (Schw.) Wolf. [Polyporaceae; Poria.] 15g  ·Atractylodes macrocephala Koidz. [Apiaceae Lindl.; Atractylodis Macrocephalae Rhizoma.] 15g  ·Rhizome of Manyleaf Paris. [Liliaceae Juss.; Paris polyphylla Smith var.chinensis （Franch.）Hara.] 15g  ·Rehmannia glutinosa Libosch. [Scrophulariaceae Juss.; Rehmanniae Radix Praeparata.] 10g  ·Angelica sinensis (Oliv.) Diels. [Apiaceae Lindl.; Angelicae Sinensis Radix.] 10g  ·Citrus medica L. var. sarco- dactylis Swingle. [Rutaceae Juss.; Citri Sarcodactylis Fructus.] 10g  ·Paeonia lactiflora Pall. [Paeoniaceae; Paeoniae Radix Alba.]10g  ·Cremastra appendiculata (D.Don) Makino. [Orchidaceae; Cremastrae Pseudobulbus Pleiones Pseudobulbus.] 10g  ·Prunella vulgaris L. [Lamiaceae Martinov; Prunellae Spica.] 20g | N | N |
| Zhang 2015 | Modeified Xiaoyao Powder | - | ·Bupleurum chinense DC. [Apiaceae Lindl.; Bupleuri Radix.] 15g  ·Angelica sinensis (Oliv.) Diels. [Apiaceae Lindl.; Angelicae Sinensis Radix.] 15g  ·Atractylodes macrocephala Koidz. [Apiaceae Lindl.; Atractylodis Macrocephalae Rhizoma.] 12g  ·Poria cocos (Schw.) Wolf. [Polyporaceae; Poria.] 15g  ·Paeonia lactiflora Pall. [Paeoniaceae; Paeoniae Radix Alba.] 12g  ·Ziziphus jujuba Mill. var. spinosa (Bunge) Hu ex H. F. Chou. [Rhamnaceae Juss.; Ziziphi Spinosae Semen.] 30g  ·Gardenia jasminoides Ellis. [Rubiaceae Juss.; Fructus Gardeniae Preparatus.] 9g  ·Salvia miltiorrhiza Bunge.[Lamiaceae Martinov; Salviae Miltiorrhizae Radix et Rhizoma.] 20g  ·Vatica mangachapoi Blanco.[Rutaceae Juss.; Green Tangerine Peel.] 12g  ·Glycyrrhiza uralensis Fisch. [Fabaceae Lindl.; Glycyrrhizae Radix et Rhizoma Praeparata Cum Melle.] 6g | N | N |
